# Fatal Retroperitoneal Bleeding Caused by Metastasis of a Sigmoid Carcinoma

**DOI:** 10.1155/2011/373047

**Published:** 2011-08-16

**Authors:** Cornelis G. Vos, Arjan W. J. Hoksbergen

**Affiliations:** Department of Surgery, VU University Medical Center, 1081 HV Amsterdam, The Netherlands

## Abstract

Retroperitoneal bleeding is relatively rare and a potentially life-threatening condition with significant mortality. Early recognition requires a high index of suspicion. Increased life expectancy, the widespread use of anticoagulants, and the rise of endovascular interventions have caused an increase in the incidence of retroperitoneal bleeding. We present a case of a 74-year-old woman who died because of retroperitoneal bleeding caused by retroperitoneal metastasis of a sigmoid carcinoma with angioinvasive growth into a lumbar artery. In addition we discuss etiology, diagnostic management, and treatment strategy.

## 1. Introduction

Retroperitoneal bleeding is relatively rare, and recognition requires a high index of suspicion [1]. It is a potentially life-threatening condition with a mortality rate of up to 20% [2]. 

The widespread use of anticoagulants and the increased life expectancy cause an increase in the incidence of retroperitoneal bleeding [3, 4]. In addition, the rise of endovascular interventions causes an increase in the incidence of (iatrogenic) retroperitoneal bleeding as well. The cause of a retroperitoneal bleeding is sometimes readily identified, especially in the case of iatrogenic bleeding or retroperitoneal bleeding due to trauma. However, in other cases the underlying cause is difficult to find.

We report a unique case of a fatal retroperitoneal bleeding caused by a metastasis of a sigmoid carcinoma. Additionally, an overview of all causes of retroperitoneal bleeding, published in Medline between 1950 and 2010, is given and contemporary management is discussed.

## 2. Case Presentation

A 74-year-old female was presented to our emergency department with fever, malaise, and intermittent left abdominal pain. The medical history included resection of a T_3_N_2_M_0_ sigmoid carcinoma, 4 months before admission. Medical examination showed a pale female with a breathing frequency of 32 breaths/minute, a blood pressure of 90/40 mmHg, and a pulse rate of 140 beats/minute. The temperature was 38.9 degrees Celsius. She had no signs of peritonitis. Laboratory testing showed the following (normal range between parentheses): hemoglobin 4.7 mmol/L (7.5–10.0 mmol/L), CRP 362 mg/mL (<10 mg mg/mL), leucocytes 41.3 × 10^9^/L (4.0–10.0 × 10^9^/L), creatinine 629 mmol/L (55–90 mmol/L), and urea 47.6 mmol/L (2.5–7.5 mmol/L). A chest X-ray showed no abnormalities. Urinalysis was positive for nitrites and leukocytes, and microscopy demonstrated bacteria. Abdominal ultrasound demonstrated hydronephrosis of the left kidney, and nephrostomy tube drainage was performed.

**Figure 1:**
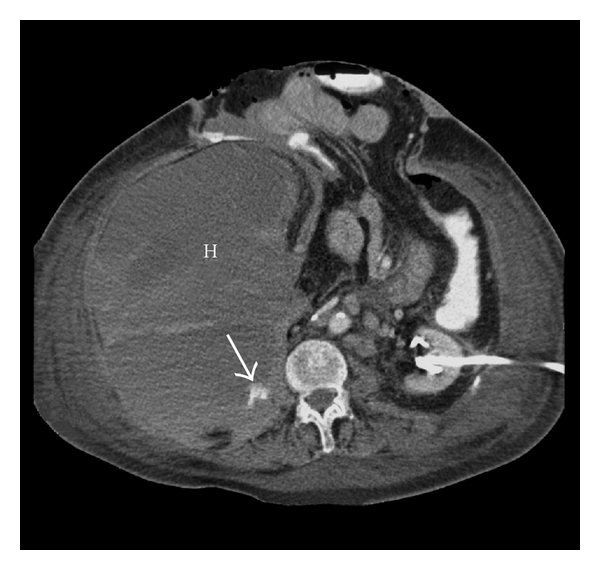
CT angiography demonstrating a large retroperitoneal hematoma (H) with evident mass effect and contrast extravasation (arrow).

**Figure 2:**
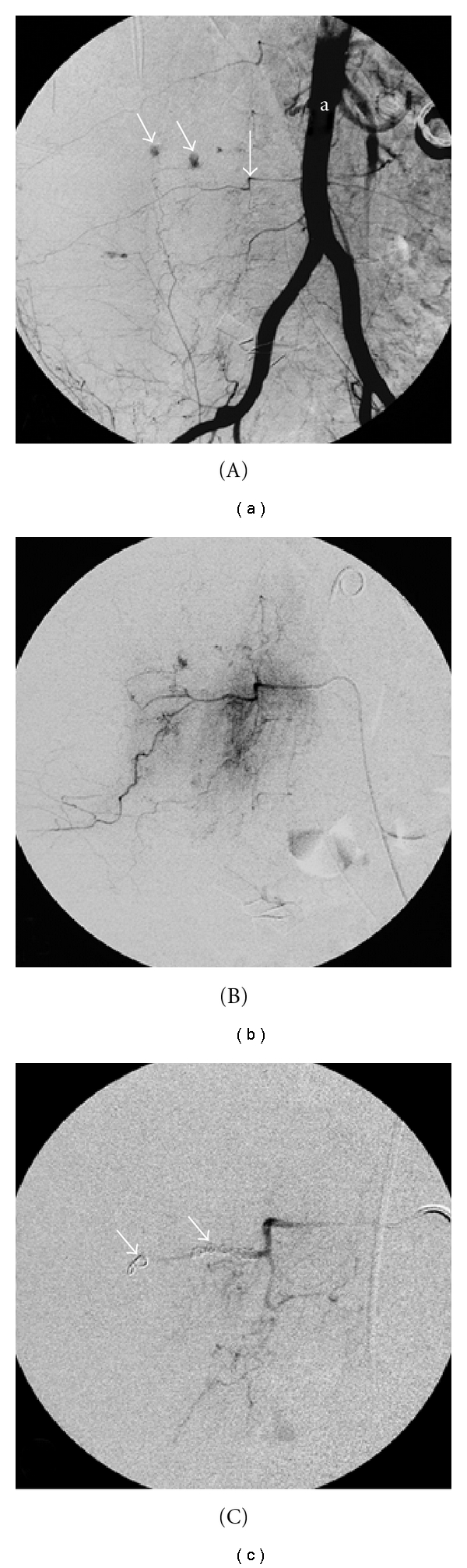
Angiography with coil embolisation; (A) overview of the aorta (a), the bleeding right lumbar artery (arrow) and 2 sites of active bleeding (thin arrows); (B) detailed view of the bleeding lumbar artery with contrast extravasation; (C) coil embolisation (arrows) of the bleeding right lumbar artery.

She was admitted to the intensive care unit with the diagnosis of urosepsis. Vital functions were stabilized, antibiotic treatment was started, and she was treated with central veno-venous hemofiltration (CVVH) because of renal failure. During CVVH, intravenous heparin was routinely administered. Initially the patient recovered, but on day 6 she developed shock with swelling of the abdomen and a sudden fall in hemoglobin count from 5.6 to 2.5 mmol/L (7.5–10.0 mmol/L) without signs of gastrointestinal bleeding. Because of severe hemodynamic instability, urgent laparotomy was performed, but no source of bleeding was identified. In the next 12 hours, the patient remained hemodynamically unstable and the laparotomy wound started to leak serosanguineous fluid. At relaparotomy this time a large retroperitoneal hematoma was found. Because there was no intraperitoneal bleeding and the hematoma was confined to the retroperitoneal space, the hematoma was left untouched and the abdomen was closed again. Urgent CT-scanning demonstrated a large retroperitoneal hematoma with a paralumbar blush (Figure 1). Catheterization of the femoral artery, and subsequent selective coil embolisation of a bleeding right lumbar artery was performed (Figure 2). However, despite intensive therapy, the patient remained hemodynamically unstable and died 6 hours later.

On autopsy a metastasis of the previously removed sigmoid carcinoma was identified. On microscopy the metastasis demonstrated angioinvasive growth in the right lumbar artery that had been coiled. A second, para-aortic metastasis was found which compressed the left ureter causing the hydronephrosis leading to the patient's presenting problem of urosepsis.

## 3. Discussion

Retroperitoneal bleeding caused by a metastasis of a sigmoid carcinoma has not been previously described. In this case the patient presented with urosepsis and subsequent renal failure, which was caused by a second metastasis, which compressed the left ureter and caused hydronephrosis. The heparin, administered during CVVH, might have intensified or prolonged the retroperitoneal bleeding caused by the metastasis in the right lumbar artery.

In the current literature (Medline 1960–2010, no restrictions on language), only 12 cases of retroperitoneal bleeding due to metastases are described. These were retroperitoneal metastases of the following primary tumors: non-small-cell lung carcinoma (*n* = 7), small cell lung carcinoma (*n* = 1), choriocarcinoma (*n* = 1), hepatocellular carcinoma (*n* = 1), chondrosarcoma of the rib (*n* = 1), and gastric sarcoma (*n* = 1) [5–16].

A wide range of causes of retroperitoneal bleeding have been described. It includes trauma, iatrogenic causes, malignancies of the kidneys or adrenal glands, vascular pathology (i.e., aneurysms or dissection), and coagulation disorders (see Table 1). When the cause of spontaneous retroperitoneal bleeding is identified, it concerns mostly renal tumors (57%–63%), vascular pathology (18%–26%), and inflammatory diseases (11%) [17]. Furthermore, the increase in the number of endovascular interventions leads to more iatrogenic retroperitoneal bleedings, especially when access is gained through the femoral artery [2]. However, frequently a specific cause is never found. These retroperitoneal hematomas are mostly found in patients who use anticoagulant therapy or have coagulation disorders. The incidence of retroperitoneal bleeding is 0.6–6.6% in patients using anticoagulants. However, in patients without any anticoagulant usage, retroperitoneal bleeding is rare [2].

The exact pathophysiology and pathogenesis of spontaneous retroperitoneal bleeding remain unclear. It has been hypothesized that occult vasculopathy and arteriosclerosis can make arteries vulnerable. A subsequent microtrauma, for example, occurring during coughing or sports, might cause a rupture of these vulnerable arteries. However, histopathologic evidence for this theory has not been found so far [2].

Retroperitoneal bleeding should be suspected in high-risk patients (high age, use of anticoagulants) who present with anemia or signs of shock without an evident source of bleeding, such as gastrointestinal blood loss. In the management of retroperitoneal bleeding, it is important to differentiate between hemodynamically stable and hemodynamically unstable patients. Stable patients can be managed conservatively by withdrawal of anticoagulant therapy, correction of coagulopathy, fluid resuscitation, and blood transfusion, if necessary [1, 2].

Hemodynamically unstable patients need further investigation and intervention to stop the bleeding. Ultrasonography is unreliable for demonstrating a retroperitoneal hematoma. A CT scan is the golden standard for detecting and locating a retroperitoneal hematoma. In case of profound bleeding, such as in this case, contrast extravasation can even be visualized. When the bleeding site is located, endovascular treatment by selective coil embolisation can be performed. Alternatively, a covered stent can be placed in the bleeding artery, under the condition that the diameter of the vessel permits this approach. For example, in the case of a bleeding puncture hole in the distal external iliac artery, caused by a percutaneous endovascular procedure through the groin. When no contrast extravasation is demonstrated by CT, there is low probability that the bleeding focus will be visualized during angiography. However, when the site of bleeding is surmised based on clinical information (e.g., trauma mechanism, prior surgery, endovascular procedure, or presence of tumor), selective arterial catheterization might allow visualization and subsequent treatment of the bleeding vessel. 

When endovascular intervention is not possible or available and the patient remains hemodynamically unstable despite aggressive conservative treatment (as described above), surgery can be indicated. A disadvantage of surgical therapy is the fact that the retroperitoneal space is opened, and a possible tamponade effect will be disturbed. However, any visible active bleeding can be controlled, and the hematoma can be evacuated during surgery. In case of diffuse bleeding, the retroperitoneum can be packed and re-explored after 24 to 48 hours [18]. Another indication for open surgery is the development of an abdominal compartment syndrome due to a large retroperitoneal hematoma [19, 20]. Percutaneous drainage of such hematomas has been described and might be useful in case of femoral nerve compression or abdominal compartment syndrome. However, the pressure reduction after drainage might cause rebleeding in the hematoma. This procedure is therefore not indicated in the acute phase of bleeding [1]. 

## 4. Conclusion

Retroperitoneal bleeding has a wide range of possible causes. A high index of suspicion is required to identify this potentially lethal condition. This is the first case describing a retroperitoneal bleeding caused by a metastasis of a sigmoid carcinoma that invaded a lumbar artery. In patients with a sudden fall in hemoglobin count and who belong to a high-risk group (high age, anticoagulant therapy, recent endovascular intervention), retroperitoneal bleeding should be considered, when no other cause of bleeding can be identified. CT angiography is the golden standard for the identification of the hematoma and possible bleeding focus. It also allows evaluation of endovascular treatment options. Therapy may consist of conservative, endovascular, or surgical treatment, depending on the cause of bleeding and the hemodynamic state of the patient.

##  Conflict of Interests

The authors declare no conflict of interests.

## Figures and Tables

**Table 1:** Reported causes of retroperitoneal bleeding (Medline 1950–2010).

*Trauma*

*Iatrogenic*
Femoral catheterization
Central line insertion
Perforation v. cava + aorta by v. cava filter
Erosion aorta by spine fixation material

*Vascular*

*Aneurysms*
Aorta
Renal artery
Adrenal artery
Uterine artery
Pancreaticoduodenal artery
Ovarian artery
*Dissection*
Ovarian artery
Inferior mesenteric artery
*Spontaneous rupture*
Portal vein
Splenic vein
Superior mesenteric artery
Uterine artery (at labor)
Iliac vein
*Other vascular causes*
Fibromuscular dysplasia of common iliac artery
Arteriovenous malformation

*Miscellaneous*

Idiopathic
Pancreatic pseudocyst
Varicosis due to liver cirrhosis
Iliopsoas muscle bleeding
Acute cholestatic viral hepatitis
Dilatation v. cava due to right heart failure.
Neurofibromatosis type I

*(Ad)renal*

*Tumors*
Angiomyolipoma
Phaeochromocytoma
Renal cel carcinoma
*Infections*
Abces
Pyelonefritis
Tuberculosis
*Miscellaneous*
(ad)renal cysts
Renal infarction
Nephritis/nephrosclerosis
Lymfangioma
Transplant rejection
Transplant rupture
Kidney stones

*Malignancies*

*Tumors*
Thymoma
Gastric carcinoma
*Metastasis*
NCSLC
Choriocarcinoma
HCC
Small cell lung carcinoma
Chondrosarcoma
Gastric sarcoma

*Systemic*

*Anticoagulants *
*Vasculitis*
*Hematologic disorders*
Leukemia
Polycythemia
Hemophilia
ITP
*Alcohol and NSAID use *
*Amyloidosis*
